# Supplementary material for: Healthcare workers’ perspectives on the availability and use of mobile health technologies for disease diagnosis and treatment support in the Ashanti Region of Ghana
Source: PLoS One. 2024 Apr 16;19(4):e0294802. doi: 10.1371/journal.pone.0294802 (PMC11020861; doi:10.1371/journal.pone.0294802)
Supplement: S1 Table — (DOCX) [file pone.0294802.s001.docx]

S1 Table : The code book of data analysis conducted in NVivo software

Nodes

| Name | Description | Files | References |
| --- | --- | --- | --- |
| Acceptance of using mHealth apps |  | 1 | 1 |
| Impressions on mHealth for disease screening |  | 1 | 1 |
| mHealth is safe |  | 2 | 2 |
| mHealth very useful |  | 10 | 10 |
| public attitude |  | 1 | 1 |
| Impressions on mHealth for treatment procedures |  | 1 | 1 |
| mHealth good for treatment |  | 3 | 3 |
| mHealth is helpful |  | 10 | 10 |
| perception of mHealth for disease screening |  | 1 | 1 |
| mHealth is helpful |  | 12 | 13 |
| mHealth speeds up work |  | 3 | 3 |
| perception of mHealth for treating conditions |  | 1 | 1 |
| mHealth is useful |  | 12 | 14 |
| Readiness of the current health systems |  | 1 | 1 |
| high level of tolerance |  | 2 | 2 |
| low awareness level |  | 1 | 1 |
| makes work easier |  | 3 | 3 |
| mHealth on covid 19 cases |  | 1 | 1 |
| screening |  | 4 | 4 |
| treatment |  | 9 | 9 |
| paperless system |  | 2 | 2 |
| willingness to accept mHealth |  | 6 | 6 |
| challenges in using mHealth |  | 1 | 1 |
| conflicting information sources |  | 2 | 2 |
| mHealth apps not localized |  | 1 | 1 |
| Cost of implementing mHealth applications |  | 1 | 1 |
| expensive |  | 14 | 14 |
| high cost of data |  | 5 | 5 |
| internet connectivity |  | 7 | 8 |
| poor mobile network |  | 3 | 3 |
| lack of education |  | 4 | 4 |
| Institutional culture |  | 2 | 2 |
| Unawareness |  | 4 | 5 |
| willingness of health workers |  | 1 | 1 |
| stable power supply |  | 2 | 2 |
| unavailability of mHealth devices at the facility level |  | 4 | 4 |
| mHealth availability for treatment |  | 1 | 1 |
| available at facility level |  | 4 | 4 |
| available at personal level |  | 13 | 13 |
| mHealth availability for disease screening |  | 1 | 1 |
| available at facility level |  | 3 | 3 |
| available at personal level |  | 13 | 14 |
| mHealth complementing healthcare delivery |  | 1 | 1 |
| mHealth is supportive |  | 6 | 6 |
| Useful |  | 8 | 8 |
| Mobile health technology |  | 1 | 1 |
| mobile phones |  | 12 | 12 |
| System |  | 2 | 2 |
| Required skills and training |  | 1 | 1 |
| basic personal knowledge in technology |  | 8 | 8 |
| formal training |  | 8 | 8 |
| No formal training |  | 7 | 7 |
| strategies to strengthen the use of mHealth apps |  | 1 | 1 |
| Factors to consider in scaling-up mHealth apps |  | 1 | 1 |
| stable internet connectivity |  | 3 | 3 |
| internet connectivity |  | 4 | 4 |
| internet connectivity (2) |  | 3 | 3 |
| strengthening the use of mHealth |  | 1 | 1 |
| develop standard treatment guidelines in a local app form |  | 1 | 1 |
| common protocol |  | 1 | 1 |
| credible information |  | 1 | 1 |
| develop apps locally |  | 1 | 1 |
| localizing information base |  | 1 | 1 |
| mHealth policy |  | 1 | 1 |
| provision of logistics |  | 12 | 12 |
| affordable data |  | 2 | 2 |
| logistics |  | 6 | 6 |
| procuring mHealth devices |  | 8 | 8 |
| structured standard treatment guidelines |  | 1 | 1 |
| education |  | 3 | 3 |
| educating health workers and patients |  | 6 | 6 |
| sensitizing health managers and professionals |  | 3 | 3 |
| training health workers |  | 5 | 5 |
| training of health workers |  | 4 | 4 |
