# Supplementary material for: Healthcare workers’ perspectives on the availability and use of mobile health technologies for disease diagnosis and treatment support in the Ashanti Region of Ghana
Source: PLoS One. 2024 Apr 16;19(4):e0294802. doi: 10.1371/journal.pone.0294802 (PMC11020861; doi:10.1371/journal.pone.0294802)
Supplement: S2 Table — (DOCX) [file pone.0294802.s002.docx]

**Supplementary file 1:** Interview guide

University of Kwazulu-Natal, Durban, School of Nursing and Public Health, Discipline of Public Health Medicine

**Title:** Mobile health (mHealth) technology for disease diagnosis and treatment support by health professionals in Ghana

**Main objective:** *To explore the perspectives of health professionals on the use of mHealth for disease diagnosis and treatment support in Ghana*.

| **Introduction**: "Hello, my name is Ernest Osei. I will conduct the interview, record and take notes. I invited you to discuss the availability and use of mobile health (mHealth) technologies for disease diagnosis and treatment support and your perceptions of the implementation of mHealth interventions.  I will ask you several open questions. Your personal opinions and views are very important to us. There are no right or wrong answers. Please feel welcome to express yourself during the interview.  This conversation will be recorded on tape. This is only for the purpose of our research, only the lead researcher indicated on the consent form (and I) will listen to the tape. No names or personal information will be used in the report.  The interview will last for about 40 minutes. I hope everything is clear about the course of the interview. | | | |
| --- | --- | --- | --- |
| Number | | Question | |
| 1. | | Please, what do you know about mobile health technology. | |
| 2. | | Do you share the opinion that mHealth devices are readily available to health workers for disease screening in your facility.  What is your opinion on the availability of mHealth devices to health workers for use to support treatment procedures in this facility? | |
| 3. | | Do you share the opinion that health professionals have the required training on how to use mHealth devices for disease screening.  Would you also say that as health professional you have the requisite the skills on how to use mHealth devices for supporting treatment procedures? | |
| 4. | | What is your perception towards the current use of mHealth devices for disease screening in your facility. | |
|  | What is your opinion on the current use of mHealth devices for treatment procedures of patients' conditions in this facility?  What do you think must be strengthened to encourage the continuous use of mHealth applications?  What is your opinion on the challenges with the current use of mHealth devices for screening of diseases  What is your perceptions on the challenges with the current use of mHealth devices for treatment procedures  What is your perception on the current health system's readiness to accommodate mHealth applications for disease screening and treatment procedures of patients' conditions  What is your opinion on how mHealth applications will complement the current healthcare delivery procedures |  |  |
| 5. | Given your experience with what mHealth serves in this facility, what do you think needs to be set up first before scaling-up mHealth applications on a large scale.  What is your perception of costs associated with the implementation of mHealth applications by healthcare authorities, and other donor partners in this facility? |  |  |
| 6. | What are your general impressions on the use of mHealth devices for disease screening services in your facility.  What are your impressions on the use of mHealth devices for treatment support in your facility. |  |  |
| 7. | Do you share the opinion that mHealth devices or applications have been used to support screening and treatment procedures of the current COVID 19 outbreak |  |  |
| 8. | In conclusion, what do you suggest to the Ministry of Health or Ghana Health Service, or health facilities on how to scale-up mHealth activities for screening.  What suggestions do have for GHS on how to scale-up mHealth activities for treatment support? |  |  |

Thanks for your participation and cooperation
