# Supplementary material for: Healthcare workers’ perspectives on the availability and use of mobile health technologies for disease diagnosis and treatment support in the Ashanti Region of Ghana
Source: PLoS One. 2024 Apr 16;19(4):e0294802. doi: 10.1371/journal.pone.0294802 (PMC11020861; doi:10.1371/journal.pone.0294802)
Supplement: Attachment — Submitted filename: Response to Reviewers-revised.docx [file pone.0294802.s003.docx]

**Author’s response to reviews:** Manuscript number: PONE-D-21-22485R1

**Title:** Healthcare workers’ perspectives on the availability and use of mobile health technologies for disease diagnosis and treatment support in the Ashanti Region of Ghana

Dear Reviewers

Thank you for reviewing our manuscript titled: Healthcare workers’ perspectives on the availability and use of mobile health technologies for disease diagnosis and treatment support in the Ashanti Region of Ghana and providing constructive feedback. We have considered your comments and recommendations. Below please find our responses to your comments, queries, and suggestions. All revisions have been highlighted in yellow in our main manuscript. Any further comments will be addressed and modified by us.

**Reviewer #1: Comments**

| This paper reports on 14 interviews with health workers on mhealth. It is generally clearly written, and all appropriate steps for qualitative analysis have been followed.  Two sets of comments, the first a little more substantive, the second more minor edits. More inter-related substantive comments | Thank you for your general comments. |
| --- | --- |
| More inter-related substantive comments - As it stands, the paper feels rather thin, based as it is on high level opinions on all mhealth applications. But mhealth applications and the way in which they might be applied across all potential health-related decisions and actions are hugely variable (eg accessing the internet for information, follow-up reminders for different types of disease/care etc etc) raising questions about the real value of reporting such generic viewpoints (and in the discussion of comparing them to wider literature). | Thank you for your comments. We have made a lot of significant changes in the entire manuscript. |
| I note a reference to a survey in reference 42. A suggestion would be to weave these findings if possible into the background, methods and findings (obviously ensuring proper reference to that published paper), in order to provide better context to qualitative views being shared. | This statement ‘‘This qualitative study was carried out as a follow-up to a survey study of 100 randomly chosen primary healthcare facilities in the Ashanti region of Ghana’’ has been placed right under **Materials and methods, refer to page 5, lines 118-119** |
| - There is not much evidence of critical analysis of what's being said by participants - eg re accessing info/guidance on the internet (what's being looked up and is that considered appropriate), re training (what kinds of training and is that really valuable etc).  Is what the health workers are discussing hypothetical (if they had the opportunity), or in practice (based on experience?). Possibly as above, linking the findings to the survey work would assist with interpreting/analysing the findings a little more for the reader. | Respectfully, we disagree with reviewer on this score, we have done a comprehensive analysis of what the professionals said.  For instance, on the training aspect, they are being trained on how to use mobile phones to search for quality and current information, how to search for information from mobile apps and others, these really add value to healthcare delivery. **Please, refer to page 13, lines 299-310.**  The health workers gave the information based on their experience from practice and on the field. |
| There are some editorial issues the authors should correct but generally the English is strong and clear. The authors should really carefully review all the sentences and wording.  For example first sentence in the abstract is incomplete, an error repeated again the main paper.  Also, the first sentence of intro needs clarifying - instead of 'are used', do the authors mean 'are currently more widely available and commonly used in...'?  And there are two sentences on page 5 second para that start... Although (the first sentence) followed by However (the second sentence). This is not an ideal flow.  Also SARs - all words start with a capital or none | All grammatical errors have been corrected in the entire manuscript.  This has been revised accordingly. Please, refer to the **Abstract section, lines 26-27.**  This has been amended accordingly. Please, refer to the **Introduction section, page 3, lines 55-56.**  This has been revised accordingly. **Please**, **refer to page 4, lines 103-107.**  All the necessary changes have been made. |

**Reviewer #2: Comments**

| I appreciate the effort made by the authors to describe their study. There are some worthwhile points made in the Results section, but overall, the manuscript has many language errors and text that is not understandable. It needs major re-organizing and re-writing to be clear, consistent, and useful to readers. I have added a number of specific comments and suggestions below to be helpful as the authors consider revisions. | Thank you for your general comments |
| --- | --- |
| **Abstract** 1. The Introduction of the Abstract says the study’s main aim is to identify challenges healthcare professionals face regarding the availability and use of mHealth applications for disease screening and management at point-of-care. The same point is made again on page 5, lines 132-134. The title suggests something more open. It’s important that the title and description of the study aim be consistent. Can the authors revise one of the other – title or description of the study aim? | This has been amended accordingly. **Please refer to Abstract, lines 29-31 and Introduction section page 4, lines 111-113** |
| 2. The Methods section of the Abstract says “the data were categorized into groups with the assistance of NVivo version 12. Our data was organized and condensed into minor forms before coding began….” It is not clear what “categorized into groups” means. I know space is tight in the Abstract, so perhaps take this out and then make sure it is clarified in the main body of the manuscript. | This has been clarified accordingly. Please, **refer to Abstract lines 35-36 and the main body, page 6, lines 176-177.** |
| **Introduction**  3. The first paragraph has a lot of useful information in it. It’s quite long though, so I would suggest breaking it up in the middle (roughly) to improve readability. A good place to start the next paragraph would be at line 86 “The digital landscape around the world including sub-Saharan Africa…” | This has been amended accordingly as suggested by the reviewer. The second paragraph begins from: “The digital landscape around the world including sub-Saharan Africa…” **Please, refer to page 3, lines 63-75.** |
| 4. On page 4, the paragraph beginning with “The literature revealed that healthcare professional used …” is hard to follow. It starts with how healthcare workers have used mHealth applications during the present COVID-19 era, and then goes into more general evidence on the views of healthcare workers toward mHealth tools (not related to COVID). It would be easier to follow the authors if use during COVID was described separately from the views of healthcare workers. | This has been done as suggested by the reviewer. Please, **refer to page 4, lines 92-98.** |
| 5. The authors write on page 4, in the paragraph beginning with “Considering the critical role that mHealth technologies…” that mHealth plays a “critical role” in promoting better healthcare services in SSA. However, they have not presented evidence to show this. They note that mHealth interventions are viewed as essential tools and that they represent emerging strategies for a variety of activities. They note that use of mobile phones is rising. They describe a variety of challenges providing care in Ghana and other settings, but also note that Ghana needs to build more mobile network infrastructure. They then describe the views of healthcare workers towards mHealth approaches. I do not see anything demonstrating that mHealth strategies actually have improved healthcare services in SSA. Can the authors please provide such evidence or revise this statement accordingly? | The statement ‘‘Considering the usefulness of mHealth technologies and applications in healthcare delivery in LMICs, including Ghana ’’ has been revised accordingly. **Please, refer to page 4, lines 108-109.** |
| **Materials and Methods**  6. On page 6, lines 143-44: what does “interpretive paradigm” mean? Is the next sentence meant to clarify what it means? I’m afraid it wasn’t clear to me and I found the paragraph confusing. | This has been revised accordingly**. Refer to page 5, lines 122-125.** |
| 7. The first two paragraphs in this section have overlapping and duplicative information. I would suggest putting the information describing the Ashanti region that is now in the second paragraph into the first paragraph, right after the first sentence that describes the main design. Then the authors could move all the information about the IDIs themselves into the second paragraph. This would improve the flow. | This has been amended accordingly as suggested by the reviewer. Kindly refer to the **study design and study setting section, page 5. Paragraph one moved to paragraph two and paragraph two now paragraph one.** |
| 8. There seems to be conflicting information about who was eligible. In the first paragraph under “Study population” (page 7, lines 174-176) the authors say that “All the potential participants were invited to participate in this qualitative study…” However, the next paragraph starts by saying that the research team purposively selected 14 healthcare professionals…” (lines 181-182). Can the authors please clarify how recruitment took place? | The statement “All the potential participants were invited to participate in this qualitative study…” has been removed to bring more clarity to the recruitment process. **Refer study population section, page 6.** |
| 9. The authors write in the middle of the paragraph under “Sampling and sample size” that the study was carried out as a follow up to survey (lines 185-187). This is important framing of the present study. It should go at the beginning of the Materials and Methods section and not be buried in this paragraph. | This has been removed and placed at the beginning of **Materials and methods section** as suggested by the reviewer. **Refer to page 5, lines 118-119.** |
| 10. The last sentence in the paragraph under “Sampling and sample size” on selection of the primary healthcare clinics where the study took place should go before a description of the healthcare workers to help guide the reader and help them appreciate the context of this work. | The statement ‘‘Primary healthcare clinics with the highest and lowest availability and use of mHealth for disease diagnosis and treatment support by healthcare workers were selected to participate in this qualitative study’’ has been moved as suggested. **Refer to page 5, lines 139-141.** |
| 11. Page 8, line 206: what does “We condensed the data into more minor forms” mean? | This has been revised accordingly. **Refer to page 6, lines 176-177.** |
| 12. The Ethics section on page 8 is a bit confusing. Could the authors confirm that every participant provided signed informed consent before participating in the study? | This has been revised accordingly. **Please, refer to page 7, lines 192-193.** |
| **Results**  13. In the paragraph under “Emerging themes” the authors write that six themes were identified, but only five themes seem to be described here. Please revise so that the language is consistent. (It seems from reading further into the Results that the theme “Challenges in using or implementing mHealth applications” (page 12, line 340) appears to have been left out of the description of the six main themes.) | This has been amended to reflect the six themes, thank you for your observation. **Please, refer to page 9, lines 206-210.** |
| 14. Out of respect for participants, I believe it is important that the direct statements of participants be presented without major grammatical errors. Sometimes such errors are carried over into a manuscript or report due to imperfect translation from another language into English, but here the authors say that the participants were comfortable using English. I would recommend that all direct statements be corrected so that they are grammatically correct and understandable. An example of a statement that would benefit from some slight revisions is the one on lines 248-249 on page 9: “Yes, they are mostly available because personally, most health staff have their phones, but unavailable at the facility level, in Ghana which facility will purchase mobile phones for use.” | All grammatical errors have been corrected throughout the entire manuscript. |
| **Discussion**  15. The first two paragraphs of the discussion summarize the main findings in quite a bit of detail. I think that’s not necessary. Rather, is there a way to indicate here why the study was unique or novel, or contributes something new? Perhaps this is the first such study in Ghana? | Thank you for your observation, this has been amended. Only the paragraph two summarizes the main findings of the study. **Refer to page 18, lines 444-466.** |
| 16. The third paragraph of the discussion seems to trying to connect the study findings to other research, which is really good. However, it covers a number of points and goes on for a long time. I would suggest breaking it up and focusing on only one point in the results and the associated research. This will help the flow for the reader enormously. | Thank you for your suggestion but respectfully we disagree with reviewer on the score that we should just focus on only one point from our results and try to connect them. We have, however, remove most of the points from this section. **Refer to page 19, lines 467-477.** |
| 17. The paragraph beginning on line 518 (the in-depth interviews created an opportunity…”) contain information on the strengths of the study. While I don’t believe all the points here are necessary, I would suggest moving several points here to the first paragraph of the discussion section as they help illustrate why the study is important. | This has been done as suggested by the reviewer. **Refer to page 19, lines 478-484.** |
| **Language issues**  18. There are many grammatical errors and confusing sentences or text in the manuscript. An example is the first sentence of the paragraph beginning on line 149 on page 6. The sentence is not a complete sentence.  Another example is the sentence on page 8, lines 213-214, beginning “This was done simultaneously…” This sentence is very confusing – I do not understand what it means. | This has been amended accordingly. **Refer to page 5, lines 127-128.**  This has been amended accordingly to bring more clarity. **Refer to page 7, lines 183-184.** |
