# Supplementary material for: Healthcare workers’ perspectives on the availability and use of mobile health technologies for disease diagnosis and treatment support in the Ashanti Region of Ghana
Source: PLoS One. 2024 Apr 16;19(4):e0294802. doi: 10.1371/journal.pone.0294802 (PMC11020861; doi:10.1371/journal.pone.0294802)
Supplement: Attachment — Submitted filename: Response to Reviewers.docx [file pone.0294802.s005.docx]

PLOS ONE Decision: Revision required [PONE-D-21-22485R2] - [EMID:62f8663b94d5c5b0]

**PONE-D-21-22485R2**

**JOURNAL: PLOS ONE**

**Title:** Healthcare workers’ perspectives on the availability and use of mobile health technologies for disease diagnosis and treatment support in the Ashanti Region of Ghana

Dear Reviewers

We are most grateful for reviewing our manuscript titled: Healthcare workers’ perspectives on the availability and use of mobile health technologies for disease diagnosis and treatment support in the Ashanti Region of Ghana and providing constructive feedback. We have considered your comments and recommendations.

Please find our responses to your comments, queries, and suggestions. All revisions have been highlighted in yellow in our main manuscript. Any further comments will be addressed and modified by us.

**Reviewer #3: Comments**

| I would like to thank the authors for this interesting work.  mHealth in abstract: For the first time, it should be written in full, not abbreviated. | We are most grateful.  This has been revised accordingly. Please, kindly to the **Abstract section, lines 27-30.** |
| --- | --- |
| Similar works have been done in Iran, such as: Ershad Sarabi, Roqhayeh, et al. "Role of mobile technology in Iran healthcare system: A review study." Journal of Health and Biomedical Informatics 4.4 (2018): 313-326.  Saeidnia HR, Mohammadzadeh Z, Hassanzadeh M. Evaluation of Mobile Phone Healthcare Applications During the Covid-19 Pandemic. Stud Health Technol Inform. 2021 May 27;281:1100-1101. doi: 10.3233/SHTI210363. PMID: 34042856. | We are grateful for suggesting these articles to us |

**Reviewer #4: Comments**

| Overall, the manuscript is well-written, and the revisions made, based on comments from the previous reviews, have improved it significantly.  However, I have few comments that require the authors' attention before the manuscript is accepted for publication. | We are grateful for your observations.  We have considered all the suggestions made by the Reviewer |
| --- | --- |
| 1. **Methods Section**  (i). There is lack of clarity on how the 14 healthcare professionals were recruited. On what basis were they recruited? How was the sample size of 14 determined? What sampling technique was adopted and the reasons for it? The authors should include a section on "Sample size and sampling technique" where these can be addressed. | This has been revised accordingly as suggested by the reviewer. Please, kindly refer to **Methodology section, page 6, lines 160 – 165.** |
| (ii). In line 168, the authors write "With the support of the trained researchers, the principal researcher conducted the interviews.  169 using the interview guide..." . However, there was no prior mention of any training for researchers. I suppose the authors meant to say trained "research assistants" instead of "the trained researchers" Please, clarify. | This has been clarified. Please, kindly refer to **Methodology section, page 6, lines 175 -176.** |
| 2. **Results Section**  Line 200-204: The authors summarised the age and work experience of participants in the first paragraph under Results. They could perform descriptive statistics to summarise the other demographic characteristics of the participants and include them in that paragraph. This would make it easier for readers to appreciate the characteristics of the participants and provide a general context to the results presented.  Table 1 can remain as it is as it shows the background of the individuals who are quoted in the results section. | This has been revised accordingly as suggested by the review. Please, kindly refer to the **Result section, page 8, lines 209 – 216.** |
| 3. **Language**   The authors have attempted to improve the readability of the manuscript following the suggestions and comments from the previous reviews. However, there are still some grammatical errors in the manuscript. I recommend a professional language editing prior to acceptance. Some instances are highlighted below: | We are grateful to the reviewer for this observation.  All grammatical errors have been addressed accordingly. |
| (i). **Abstract:** The first sentence in the introduction section is incomplete. It appears it can be complete by merging with the second sentence. This was pointed out in previous review but has still not been corrected. | This has been amended by merging the first and second sentences in the **abstract section, please, kindly refer to lines 27 – 30.** |
| (ii). **Line 58:** check the hyphen in "highly resourced-settings". It could be written as highly-resourced settings or "high-resource settings"; and in "low-and middle-income countries" there should be spacing between "low-" and "and" as in "low- and middle-income countries. | This has been amended and revised accordingly, **please, kindly refer to line 59.** |
| 4. Data availability statement appears incomplete. The authors mention that data is available without indicating where or how the data can be accessed. Please refer to relevant journal policy/guidelines. | This has been revised accordingly, **please, kindly refer to lines 523 - 524.** |
