# Supplementary material for: Healthcare workers’ perspectives on the availability and use of mobile health technologies for disease diagnosis and treatment support in the Ashanti Region of Ghana
Source: PLoS One. 2024 Apr 16;19(4):e0294802. doi: 10.1371/journal.pone.0294802 (PMC11020861; doi:10.1371/journal.pone.0294802)
Supplement: Attachment — Submitted filename: Response to Reviewers-latest.docx [file pone.0294802.s006.docx]

PLOS ONE Decision: Revision required [PONE-D-21-22485R3]-[EMID: 279b1db1e8cfb81a]

**PONE-D-21-22485R3**

**JOURNAL: PLOS ONE**

**Title:** Healthcare workers’ perspectives on the availability and use of mobile health technologies for disease diagnosis and treatment support in the Ashanti Region of Ghana

Dear Reviewers

We are most grateful for reviewing our manuscript titled: Healthcare workers’ perspectives on the availability and use of mobile health technologies for disease diagnosis and treatment support in the Ashanti Region of Ghana and providing constructive feedback. We have considered your comments and recommendations.

Please find our responses to your comments, queries, and suggestions. All revisions have been highlighted in yellow in our main manuscript. Any further comments will be addressed and modified by us.

**Reviewer: Comments**

| Reviewer comments are adequately addressed.  However, the portions on study participants under the results session (page 8, including the Table 1) is part of the method session.  Authors should consider moving the portion on study participants to the method session | We are very grateful to the Reviewer  Well noted  We have revised accordingly as suggested by the reviewer, kindly refer to **lines 159 – 164, pages 6- 7.** |
| --- | --- |
